# Seeing the forest in order and the trees in disorder: Environmental orderliness versus disorderliness affects the perceptual processing style

**DOI:** 10.1002/pchj.309

**Published:** 2019-07-11

**Authors:** Kaiyun Li, Huijing Yang, Xueyang Wang, Tuo Zhang, Ping Lu, Fengxun Lin

**Affiliations:** ^1^ School of Education and Psychology University of Jinan Jinan China

**Keywords:** conceptual, disorderliness, global/local processing, orderliness, perceptual

## Abstract

People attend to the same event or object by using a global or local processing style across different environments. Different physical environmental conditions, such as orderliness and disorderliness, activate different psychological states and produce different kinds of outcomes. However, previous work has rarely examined whether individuals exposed to different orderly or disorderly environments attend to the “global” or the “local” differently. Thus, in the current study, we conducted three behavioral experiments to directly examine the impact of disorder versus order cues on people's types of perceptual and conceptual processing (global vs. local). We asked participants to perform a typical Kimchi–Palmer figures task or a categorization task: with pre‐primed disorderly or orderly physical environmental pictures (Experiment 1), with basic visual pictures (Experiment 2), and imagining a real environment (Experiment 3). The results revealed that in any of the above operations, orderly experience led to global perceptual processing, whereas disorderly experience led to local perceptual processing. This difference in processing style was not influenced by the participants’ daily habits or their preference for the need for structure. However, this difference in perceptual processing style did not spill over to the conceptual processing style. These findings provide direct evidence of the effects of disorderliness versus orderliness on global versus local perceptual and conceptual processing and imply that environmental orderliness or disorderliness may functionally affect cognitive processing (i.e., how we see and think about events and objects). Thus, the findings creatively bridge several lines of research and shed light on a basic cognitive mechanism responsible for perceptions of order/disorder.

The physical environment deeply influences how we feel, think, and act. Different physical environmental conditions, such as orderliness and disorderliness, activate different psychological states and produce different kinds of outcomes. The relative position of objects within space as determined by certain patterns or rules is considered the key point to distinguish environmental orderliness from disorderliness (Lu, Zhang, Wei, & Liu, 2018). Research has found that in contrast to exposure to an orderly environment, exposure to a disorderly environment: may produce detrimental outcomes, such as negative affect, including perceived powerlessness (Geis & Ross, 1998) and distress (Cutrona, Russell, Hessling, Brown, & Murry, 2000), feeling unsafe (Perkins & Taylor, 1996), depression (Ross, 2000; Ross & Jang, 2000), and self‐reported anxiety (Tullett, Kay, & Inzlicht, 2015); may diminish a sense of meaning in life (Heintzelman, Trent, & King, 2013) and reduce self‐control and cognitive control (Chae & Zhu, 2014); and may have downstream effects on complex behaviors, such as increased delinquency, rule breaking, and criminal behavior (Keizer, Lindenberg, & Steg, 2008; Kotabe, Kardan, & Berman, 2016b). Notably, in contrast to these negative effects, researchers have recently found that a disorderly environment may also encourage a break from tradition and convention and thus stimulate creativity (including divergent and convergent dimensionality; Chen, Zheng, Xiaomeng, & YuanYuan, 2013; Cotroneo, 2014; Kim & Zhong, 2017; Vohs, Redden, & Rahinel, 2013). To the best of our knowledge, previous research on physical disorderliness and orderliness has largely neglected one of the most frequently investigated dimensions of cognitive processing: global (“forest”) versus local (“tree”) processing styles, including the perceptual and conceptual levels.

People attend to the same event or object by using global or local processing styles across different cultures, situations, and environments (Caparos et al., 2012; Förster & Dannenberg, 2010; Shigehiro et al., 2014). Operationally, global processing involves establishing spatial relationships among discrete local elements and linking them together to form a coherent global structure (forest), whereas local processing is based on selective attention to individual elements of an object or scene (trees; Kimchi, 1992; Lewis et al., 2004; Navon, 1977; Nayar, Franchak, Adolph, & Kiorpes, 2015). Evidence in cognitive, clinical, and social psychology has revealed a distinction between global and local processing. For example, in contrast to local processing, global processing has been shown to be associated with positive moods (Gasper, 2004; Gasper & Clore, 2002), lower levels of anxiety and obsessiveness (Kedem & Paz, 1990; Yovel, Revelle, & Mineka, 2005), and high power (Smith & Trope, 2006). Furthermore, global processing can stimulate higher‐level processing, such as creative thought (Friedman, Fishbach, Förster, & Werth, 2003). With regard to the impact of the environment on processing style, a recent study found that exposure to an urban environment was positively associated with global processing among the traditional Himba people (Caparos et al., 2012).

People have a strong need to perceive their environment as orderly and structured and to escape disorderliness and randomness (Fennis & Wiebenga, 2015; Heintzelman et al., 2013; Johnson, Martin, Brooksgunn, & Petrill, 2008; O'Brien, 2014). However, little work has examined the impact of orderly/disorderly environmental variations on global versus local processing—that is, whether individuals exposed to different orderly or disorderly environments attend to the forest or the trees differently. Therefore, the aim of the current study was to directly measure participants’ perceptual and conceptual processing styles when they were primed with or imagined orderly/disorderly environmental situations.

## Positive/negative affect linked to orderliness/disorderliness and global/local perceptual processing

In contrast to those of order, research has emphasized that the affective consequences of disorder are mostly negative, including perceived powerlessness (Geis & Ross, 1998) and distress (Cutrona et al., 2000), feeling unsafe (Perkins, Brown, & Taylor, 2010), depression (Ross & Jang, 2000), and self‐reported anxiety (Tullett et al., 2015). For example, early studies performing multilevel analyses of data from a representative sample of 2,482 adults aged 18–92 years in Illinois (from the 1995 Survey of Community Crime and Health) revealed various results. Geis and Ross (1998) first found that compared to people who report living in quiet, clean neighborhoods, people who report living in disordered neighborhoods have significantly higher levels of perceived powerlessness and negative consequences, including depression, anxiety, malaise, and illness. Neighborhood disorder refers to a perceived lack of order (a state of peace, safety, and observance of the law) and social control (acts to maintain order) in the community, including visible perceived social and physical cues (Skogan, 1986; Taylor & Shumaker, 1990). Ross and Jang (2000) further confirmed that individuals who report that they live in disorderly neighborhoods have high levels of fear and mistrust. Ross (2000) found that compared with residents of more advantaged neighborhoods, residents of disadvantaged neighborhoods have high levels of depression, and neighborhood disorder mediates the association between disadvantage and depression. Similarly, Cutrona and colleagues (2000) found that high ratings of neighborhood disorder were significantly related to distress. Recently, Tullett and colleagues (2015) found that randomness (disorderliness) leads to increased anxiety compared with order. Randomness (disorderliness) elicited the largest error‐related negativity, an event‐related brain potential associated with performance monitoring. Thus, these previous empirical studies have demonstrated that disorderliness evokes negative affect, whereas orderliness elicits positive affect.

Affective feelings can influence the way people see their world. An abundance of research has investigated the association between affective feelings/mood and global/local perceptual processing style. Fredrickson's “broaden‐and‐build theory” (Fredrickson, 1998, 2004; Fredrickson & Branigan, 2005) suggests that positive affect leads individuals to focus on the forest (global processing) at the expense of the tree (local processing), whereas the reverse is true for negative moods (Fredrickson & Branigan, 2005; Gasper, 2004; Gasper & Clore, 2002; Rowe, Hirsh, & Anderson, 2007; Tyler & Tucker, 1982; Uddenberg & Shim, 2015). For example, Gasper and colleagues (Gasper, 2004; Gasper & Clore, 2002) asked participants to write about a “happy and positive” or a “sad and negative” life event and then to complete the Kimchi–Palmer figure task (a typical perceptual processing task). They found that participants with sad moods were less likely than those with happier moods to classify figures on the basis of global features; that is, happier moods promoted a greater focus on the forest, whereas sadder moods promoted a greater focus on the trees. Fredrickson & Branigan (2005) asked participants to view a film that elicited amusement, contentment, neutrality, anger, or anxiety and then assessed their performance on the Kimchi–Palmer figure task. Their results verified Gasper's findings. Dijkstra, Pligt, and Kleef (2014) conducted a reverse manipulation in which processing style was measured by the Navon letter task. The participants judged 28 pictures selected from the International Affective Picture System (Lang, Bradley, & Cuthbert, 2005), which were evenly distributed along the pleasure continuum. The results revealed that participants in the global processing condition were more responsive to the pleasure dimension of stimuli than were participants in the local processing condition. Moreover, both situationally induced and chronic moods, such as trait happiness and optimism, are connected with attention to global as opposed to local structures, whereas trait depression and anxiety correlate negatively with global processing (Basso, Schefft, Ris, & Dember, 1996; Tyler & Tucker, 1982). Briefly stated, global processing style is related to positive affect, while local processing style is linked to negative affect.

Given the aforementioned experimentally verified associations of both disorderliness and local perceptual processing with negative affect, and because orderliness and global perceptual processing are connected to positive affect, we proposed the following hypothesis:

*Hypothesis 1*: Disorderliness might lead to local perceptual processing, whereas orderliness might lead to global perceptual processing.

## Contradictory evidence for the relationship between disorderliness and global/local conceptual processing

A number of cognitive theorists have suggested that perceptual processing styles spill over to conceptual tasks, such as the creative generation task (Förster & Dannenberg, 2010; Friedman et al., 2003; Friedman & Förster, 2001; Liberman & Förster, 2009). For example, Friedman et al. (2003) primed global versus local perception by asking participants to look at either the gestalt of a state map (forest) or a specific detail of the same map (tree). Participants were then asked to generate the most unusual exemplar they could think of for a number of categories (e.g., birds, colors, fruits). The results revealed that participants who looked at the map globally created more atypical exemplars than participants who looked at the map's details. In subsequent conceptual replication studies, researchers asked participants to view items on a computer screen within a small (local processing) or a larger (global processing) radius (Friedman et al., 2003) or primed them with the Navon letter task (Förster & Dannenberg, 2010). Then, the participants were asked to find a creative title for a cartoon and generate unusual uses for a brick. Data analysis revealed that more creative solutions were generated (such as “grind it up and use it as makeup”) following global priming than following local priming (such as “build a wall”; Förster & Dannenberg, 2010). Thus, these studies supported the link between perceptual processing and conceptual processing. Notably, research has established that positive moods can improve performance in conceptual tasks, such as breadth of categorization and creative thought (Isen, 1987; Isen & Daubman, 1984). Isen and Daubman (1984) adopted Rosch's (1975) categorization task and found that participants who first watched a comedy that elicited a positive mood were more likely to include fringe members (such as camels) into a certain category (such as vehicles) than participants who watched a movie about concentration camps that produced a negative mood, reflecting a broader conceptual categorization. Therefore, in line with the corresponding relationship between perceptual and conceptual processing and the research finding that positive moods stimulate global perceptual processing and broader conceptual categorization, we proposed the following hypothesis:

*Hypothesis 2*: In contrast to orderly environmental situations, disorderliness leads to local conceptual processing.

However, we should note prior findings that a disorderly environment may encourage people to seek novelty and unconventional routes that produce creative thought (Chen et al., 2013; Ritter et al., 2012; Vohs et al., 2013). For example, Vohs et al. (2013, Study 2) reported that participants who were sitting in a disorderly room developed more creative ideas about alternative uses for Ping‐Pong balls than participants who were sitting in an orderly room (Vohs et al., 2013). Chen et al. (2013) also showed that a disorderly environment has a positive influence on individuals’ performance in creative tasks, including divergent creativity tasks (brainstorming uses for newspaper) and convergent creativity tasks (remote association test; Chen et al., 2013). In a real working environment, many famous creative individuals, such as scientists, writers, artists, and Nobel Prize recipients, prefer and cultivate messy environments because these environments improve their work (Abrahamson & Freedman, 2007). One such creative person was Einstein, who is widely reported to have said, “If a cluttered desk is a sign of a cluttered mind, of what, then, is an empty desk a sign?” In addition, research in organizational psychology has shown that untidy, disorderly environments can be considered environmental contexts that encourage unconventional and open thought processes (Elsbach & Pratt, 2007). Given this evidence that disorderly environments may stimulate higher creativity, which may reflect global conceptual processing, we proposed the following hypothesis:

*Hypothesis 3*: An experience of disorderliness leads to global conceptual processing, while orderliness leads to local conceptual processing.

## The present study

Given previous studies and these three hypotheses, we conducted three behavioral studies to explore whether environmental orderliness or disorderliness makes perceptual and conceptual processing more global or local. The participants experienced environmental orderliness or disorderliness primed by the physical environment (Experiment 1), viewed basic visual order/disorder pictures (Experiment 2), or imagined themselves in an orderly/disorderly environment (Experiment 3) before they performed a perceptual and a conceptual task. Perceptual processing was measured by the well‐known Kimchi–Palmer figures task designed by Kimchi and Palmer (1982), which has been used frequently in studies of perception (Fredrickson & Branigan, 2005; Gasper & Clore, 2002; Hicks & King, 2007).

In addition, numerous studies have demonstrated that people have a fundamental need to view the world as an ordered and structured place composed of predictable cause‐and‐effect relations (Fennis & Wiebenga, 2015; Heine, Proulx, & Vohs, 2006; Kay, Whitson, Gaucher, & Galinsky, 2010; Landau et al., 2004); that is, maintaining a sense of order or structure might be ingrained in our nature. Recently, in a series of lab and field experiments, Fennis and Wiebenga (2015) found that the need for order was higher for participants in the disordered environment condition than for participants in the ordered environment condition. Some studies have proposed that the Personal Need for Structure (PNS) Scale can serve as a valid operationalization of people's differences in their chronic desire for simple structure (Rietzschel, De Dreu, & Nijstad, 2007; Shi, Wang, & Chen, 2009). The results showed that people who scored high on the PNS Scale were especially likely to organize social and nonsocial information in less complex ways, stereotype others, and complete their research requirements on time, whereas people who scored low on the PNS Scale behaved in the opposite way (Neuberg & Newsom, 1993). Furthermore, some individuals prefer to live in a clean environment, whereas others like chaos. These daily life habits might influence cognitive processing to some extent. Therefore, in the current study, we seek to examine whether individuals’ needs for simple structure and daily habits influence the relationship between environmental orderliness/disorderliness and cognitive processing at the perceptual or conceptual level.

## Experiment 1: Orderliness/disorderliness experiences primed by orderly/disorderly physical environment pictures

**Figure 1:**
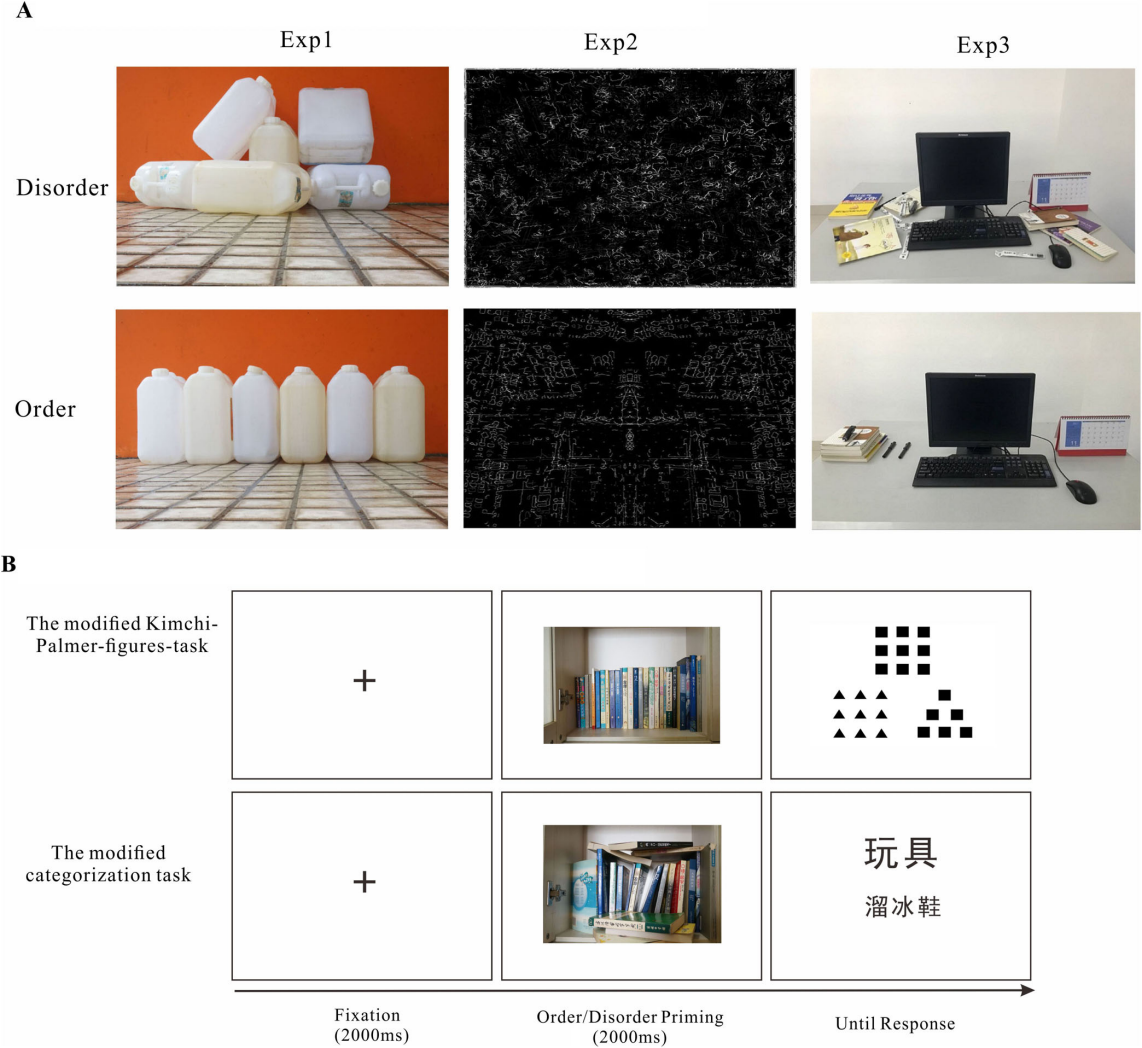
(A) Illustration of the stimuli used in the three experiments and (B) the procedure of Experiment 1. [Color figure can be viewed at wileyonlinelibrary.com]

From a cognitive perspective, Kotabe (2014) operationalized “perceived disorder or order.” Perceived disorder is an interpreted state of the world in which things are in non‐patterned and non‐coherent positions. In contrast, perceived order is an interpreted state of the world in which things are in patterned and coherent positions. Therefore, the key requirement of “physical disorder/order” is that the degree of the stimulus is processed based on orderliness, regularity and pattern and the rationality of its place. Based on Kotabe's definition, Hu (2017) created a set of pictures of a disordered physical environment by depicting places or objects artificially arranged in non‐patterns and non‐coherent patterns; conversely, the orderly physical environment images depicted places or objects artificially arranged in patterns and coherent patterns. In Experiment 1, we attempted to measure whether individuals’ perceptual and conceptual processing styles tend to be global or local after they experience orderliness and disorderliness primed by the pictures of an orderly/disorderly physical environment used in Hu's study (see the illustration in Figure 1). In addition, we examined whether individuals’ needs for simple structure and daily habits influence the relationship between the environment's orderliness/disorderliness and cognitive processing.

### Method

#### *Participants*

One‐hundred twenty‐three healthy college students were paid to participate in Experiment 1. All participants in the three experiments reported normal or corrected‐to‐normal vision. All experiments in the current study were conducted with the informed written consent of each participant and were approved by the Institutional Review Board of the School of Education and Psychology at the University of Jinan. Data from one participant were discarded because most of the response data were not recorded by the E‐prime software. Data from two participants were also discarded because the total target response time of the perceptual processing task was more than 2 *SD* from the mean. The data from the remaining 120 participants (61 females, age range: 18–22 years, *M*_age_ = 19.34 years, *SD* = 0.84 years) were used. The disorder priming condition included 61 participants’ data, and the order priming condition included 59 participants’ data.

#### *Apparatus and stimuli*

In the three experiments of the current study, the participants sat in an experiment lab that was clean and empty except for the things used in the experiments. The participants sat approximately 65 cm from a computer screen (a 19‐inch monitor, 1024 × 768 pixels, 85 Hz). They were asked to keep their heads on a headrest with their eyes focused on the center of the screen during the test session except for rest periods (of at least 1 s) between blocks. The experimental stimuli were presented using E‐prime 2.0.

##### *Orderly/disorderly physical environment pictures*

A total of 60 pictures were collected from daily life. These pictures were taken artificially based on two conditions, disorder or order, as shown in Figure 1A. A separate group of 23 participants was first shown all 60 pictures (fixed size of 600 × 398 pixels) one by one and were made aware of the content and order/disorder of the pictures.^1^ Then, they were asked to evaluate the level of order/disorder for each picture on a 7‐point Likert scale (1 = *totally disorderly*, 7 = *mostly orderly*) considering the entire range of all pictures. A paired *t* test revealed that the rating score for the degree of disorder of the disorderly pictures (*M* = 2.01, *SD* = 0.58) was significantly lower than that for the orderly pictures (*M* = 5.82, *SD* = 0.48), *t*(22) = −24.05, *p* < .001.

In addition, we asked participants to indicate the emotional responses evoked by each picture on the Self‐Assessment Manikin (SAM), which is a widely and effectively used non‐verbal pictorial assessment technique created by Bradley and Lang (1994). Participants indicated their emotional experiences in three dimensions (Pleasure, Arousal, and Dominance) on a 9‐point scale. The Pleasure scale was anchored with *very unhappy* (1 point) versus *very happy* (9 points), the Arousal scale was anchored with *very calm* (1 point) versus *very excited* (9 points), and the Dominance scale was anchored with *very submissive* (1 point) versus *very dominant* (9 points). In the measurement, the participants could place an “x” over any of the five figures in each scale or between any two figures, which resulted in a 9‐point rating scale for each dimension. Paired *t* tests revealed that for the Pleasure dimension, the orderly pictures (*M* = 6.74, *SD* = 1.60) produced significantly higher pleasantness than the disorderly pictures (*M* = 4.69, *SD* = 2.11), *t*(22) = 3.52, *p* < .01. In the Arousal dimension, the arousal level of the orderly pictures (*M* = 6.52, *SD* = 2.06) was significantly higher than that of the disorderly pictures (*M* = 5.04, *SD* = 1.84), *t*(22) = 2.77, *p* < .05. In the Dominance dimension, the dominance level of the orderly pictures (*M* = 6.61, *SD* = 1.47) was significantly higher than that of the disorderly pictures (*M* = 5.22, *SD* = 1.91), *t*(22) = 2.69, *p* < .01.

##### *The Kimchi–Palmer figures task*

Perceptual processing style was measured by the Kimchi–Palmer figures task (Kimchi & Palmer, 1982). During each trial, the participants had to indicate which of two comparison figures was more similar to a target figure. Each figure had a global form (square or triangle) consisting of local forms (squares, triangles or circles). Figure 1B shows the procedure of the modified Kimchi–Palmer figures task.

##### *The categorization task*

Conceptual processing style was measured by a categorization task (Isen & Daubman, 1984). For each trial, after order/disorder priming, the participants were asked to rate words on a 10‐point scale (1 = *definitely does not belong to the category*; 5 = *does not belong to the category but is very similar to members of that category*; 6 = *does belong to the category but is not a very good example of it*; and 10 = *definitely does belong to the category*). Ratings above five signal inclusion in the category. Twelve exemplar words were presented for each of four categories (vehicles, weapon, toy, furniture) based on Rosch's (1975) norms: four typical exemplars (e.g., car, handgun, dolls, and sofa), four moderate exemplars (e.g., airplane, axe, roller‐skates, lamp), and four atypical exemplars (e.g., camel, shoes, animal, and telephone). This approach measured the inclusiveness of the categorization.

##### *PNS Scale*

The cognitive structure level of the participants was measured by the Chinese version of the PNS Scale, which included 11 items (Yang, Yunhui, Lei, & Juniqi, 2008). All 11 items met the psychometric criteria. The internal consistency reliability and the split‐half reliability were all above .80. The test–retest reliability was .79. Participants were asked to rate their agreement on a 6‐point scale (1 = *disagree totally*; 6 = *agree totally*). A higher score represents a higher cognitive structure of the participants.

##### *Habits questionnaire*

Two statements were used to measure the participants’ habits: “Your living environment (room, dormitory, etc.) is messy and you don't care” and “You will keep your living environment (dormitory, desk, bed, etc.) in order.” Participants were asked to rate their agreement on a 6‐point scale (1 = *disagree totally, 6 = agree totally*). The score for the first question was reversed. The higher the average score of these two questions, the more orderly the habits of the participants tended to be.

#### *Design and procedure*

Experiment 1 was a between‐subject design with two levels: priming of the disorderly physical environment picture condition and priming of the orderly physical environment picture condition. The response time and global target scores (the number of times that participants matched the shapes on the basis of their global form rather their local details) of the Kimchi–Palmer figures task and the rating scores of the categorization task were the dependent variables. The scores of the PNS Scale and the habits questionnaire were the covariate variables.

In Experiment 1, each participant sequentially completed the Kimchi–Palmer figures task and the categorization task by using the E‐prime software and then completed three questionnaires: the behavior identification form, the PNS Scale, and the habits questionnaire.

For the Kimchi–Palmer figures task (see Figure 1B), each trial began with a central fixation cross (angle 0.8°) for 2000 ms, which was followed by a disorderly (or orderly) priming picture that was shown for 2000 ms. Participants were asked to passively view the priming picture. Then, a Kimchi–Palmer figure was presented, and participants were asked to choose which comparison figure was more similar to the target figure by pressing the “F” or the “J” button as quickly as possible. The next trial began after a blank screen was presented for 1000 ms. After six practice trials, the participants completed 48 experimental trials in which 24 disorder (or order) priming pictures were presented twice and randomly. Global processing style was measured as the number of times that participants matched the shapes on the basis of their global form rather their local details.

After the participants completed the Kimchi–Palmer figures task, there was a 5‐min rest. Then, the participants performed the categorization task. For the categorization task, each trial began with a central fixation cross (angle 0.8°) for 1000 ms, which was continually followed by two disorder (or order) priming pictures, with each picture shown for 2000 ms (see Figure 1B). A category word (vehicles, weapon, toy, furniture) and an exemplar word were then presented on the same screen, and the participants were asked to rate the inclusion of the exemplar word into the category word on a 10‐point scale by clicking a number (1–10). There were 12 trials in each condition.

Finally, the participants completed three paper questionnaires: the behavior identification form, the PNS Scale, and the habits questionnaire.

### Results and discussion

#### *Perceptual processing style*

**Figure 2:**
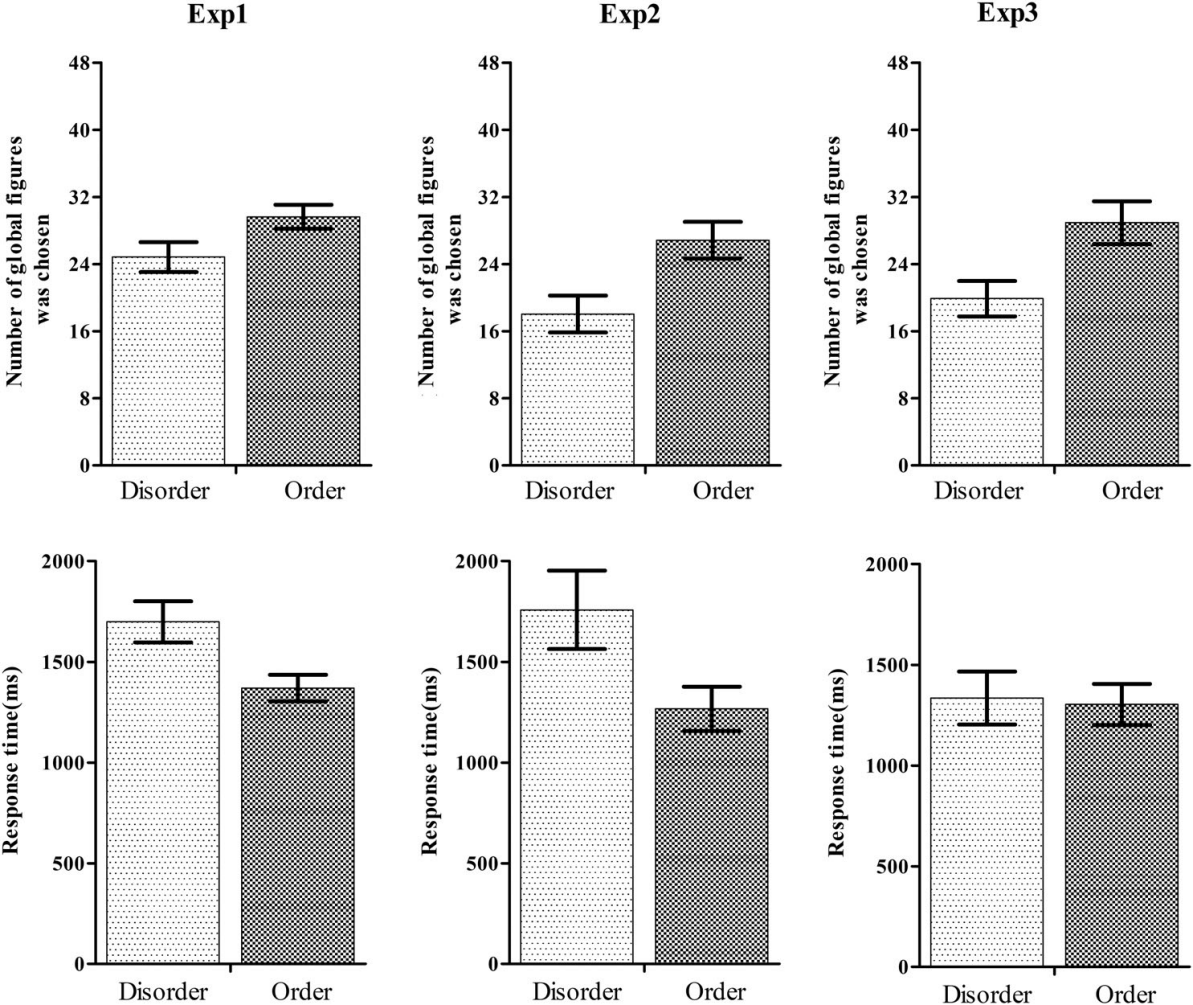
Participants’ response times and scores for the global target figure of the Kimchi–Palmer figures task in Experiments 1–3. Error bars represent 2 *SEM*.

First, an independent *t* test analysis between the disorder priming condition and the order priming condition was conducted separately for the response time and global target scores, and the Greenhouse–Geisser correction was applied to compensate for possible effects of nonsphericity in the measurements. The statistical analysis showed that the average response time in the disorder priming condition (*M* = 1699.34 ms, *SD* = 792.90) was significantly longer than that in the order priming condition (*M* = 1369.10 ms, *SD* = 512.55), *t*(118) = 2.70, *p* < .01, Cohen's *d* = 0.49. The statistical analysis of the global target scores revealed a significant difference between the two conditions, *t*(118) = −2.07, *p* < .05, Cohen's *d* = 0.38, in which the average global target score in the disorder priming condition (*M* = 24.85, *SD* = 14.03) was significantly smaller than that for fruits only (*M* = 29.63, *SD* = 11.06). Figure 2 depicts the response times and global target scores.

The Pearson correlation analysis revealed no significant correlations between the global target scores and habits and the PNS score (*r*_habit_ = .012; *r*_PNS_ = −.07). We then considered the PNS score and the habits questionnaire as covariate variables simultaneously. The univariate covariance analysis of the global target scores revealed a significant difference between the two conditions, *F*(1, 115) = −3.91, *p =* .05, $\eta_{p}^{2}=.33$, in which the average global target score in the disorder priming condition (*M* = 24.92, *SD* = 14.03) was significantly smaller than that in the order priming condition (*M* = 29.59, *SD* = 11.06). These results revealed that participants’ habits and the degree of their PNS did not influence their perceptual processing.

#### *Conceptual processing style*

**Figure 3:**
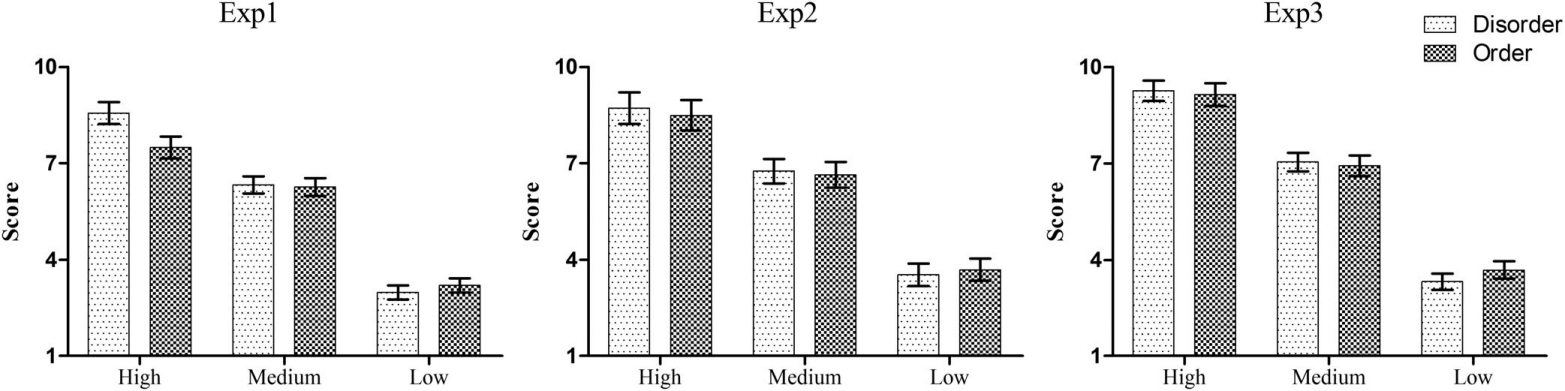
Rating scores of exemplar words of the categorization task in Experiments 1–3. Error bars represent 2 *SEM*.

A two‐way repeated‐measures analysis of variance (ANOVA) with prime type (disorder vs. order) as the between‐subject factor and exemplar type (typical exemplar vs. moderate exemplar vs. atypical exemplar) as the within‐subject factor was conducted. The analysis revealed no main effect of priming type (*p* > .05). A high main effect of exemplar type, *F*(2, 236) = 267.15, *p <* .0001, $\eta_{p}^{2}=.6$, was observed, with higher rating scores for the typical exemplar words (*M* = 8.03, *SD* = 2.68) than for the moderate exemplar words (*M* = 6.28, *SD* = 2.13) and the atypical exemplar words (*M* = 3.09, *SD* = 1.67), *p*s < .001. There was a significant interaction effect of priming type by exemplar type, *F*(2, 236) = 4.85, *p* < .001, $\eta_{p}^{2}=.039$. Further simple comparisons revealed that only at the typical exemplar level, the rating score at the disorder priming condition (*M* = 8.56, *SD* = 2.41) was significantly higher than that at the order priming condition (*M* = 7.49, *SD* = 2.87), *p* < .05. Figure 3 depicts the rating scores of the exemplar words.

The Pearson correlation analysis revealed no significant correlations between the exemplar word scores and habits and the PNS score. We further used the score of the PNS Scale and the habits questionnaire as covariate variables. The multivariate covariance analysis revealed no main effect of picture priming type (*p* > .05). A high main effect of exemplar type, *F*(2, 230) = 4.64, *p* < .05, $\eta_{p}^{2}=.039$, was observed, with higher rating scores for the typical exemplar words (*M* = 8.03, *SD* = 2.68) than for the moderate exemplar words (*M* = 6.28, *SD* = 2.13) and the atypical exemplar words (*M* = 3.09, *SD* = 1.67), *p*s < .001. There was a significant interaction effect of priming type by exemplar type, *F*(2, 230) = 4.76, *p* < .001, $\eta_{p}^{2}=.04$. Further simple comparisons revealed that only at the typical exemplar level, the rating score for the disorder priming condition (*M* = 8.56, *SD* = 2.41) was significantly higher than that for the order priming condition (*M* = 7.49, *SD* = 2.87), *p* < .05. These results revealed that participants’ habits and the degree of their PNS did not influence their type of conceptual processing.

Through the modified Kimchi–Palmer figures task, Hypothesis 1 was supported: orderly physical environment pictures lead to global perceptual processing (higher global target scores), whereas disorderly physical environment pictures lead to local perceptual processing. It might be that the higher positive pleasantness of the orderly pictures contributes to global perceptual processing, in accordance with previous claims that positive affect leads individuals to focus on the forest (global processing) at the expense of the tree (local processing), whereas for negative moods, the reverse is true (Fredrickson & Branigan, 2005; Gasper, 2004; Gasper & Clore, 2002; Rowe et al., 2007; Tyler & Tucker, 1982; Uddenberg & Shim, 2015). The results of the modified categorized task revealed that at the typical exemplar word level, the rating score for the disorderly pictures priming condition was significantly higher than that for the orderly pictures priming condition, which seems to partly support Hypothesis 3: Because a disorderly environment stimulates creative thinking (Chen et al., 2013; Vohs et al., 2013), disorderliness might lead to global conceptual processing, while orderliness might lead to local conceptual processing. The participants’ PNS scores and individuals’ habits of orderly or disorderly living did not influence the types of global and local perceptual and conceptual processing.

## Experiment 2: Orderliness/disorderliness experiences primed by basic visual order/disorder pictures

In the real world, a scene of an environment usually contains low‐level visual features (e.g., edges, colors, spatial frequency) and high‐level semantic features (e.g., places and objects; Oliva, [Link]; Oliva & Schyns, 2000; Oliva & Torralba, 2001, 2006; Rosch & Mervis, 1975). The aforementioned “physical environmental disorder/order” stimuli involved high‐level scene or semantic information. Kotabe, Kardan, and Berman (2016a) and Kotabe et al. (2016b) recently proposed a method to distinguish basic visual cues from high‐level cues in physical environmental disorder/order. They defined *visual disorder* as the perception of disorder attributable to basic (or low‐level) visual features (i.e., spatial and color features, basic visual disorder cues; Kotabe et al., 2016a, 2016b). Adopting a principled approach to reconstruct stimuli that contrasted in terms of visual disorder but lacked scene‐level or semantic (referring to meaningful information, such as that involved in the recognition of objects, places, and general descriptors) social disorder cues, they found that spatial features (e.g., non‐straight edges, asymmetry) were most important for visual disorder/order (see Figure 1A). Kotabe et al. (2016a) further reported that even basic visual disorder cues (simple spatial perceptual properties of the environment) could affect complex behavior (e.g., cheating, rule breaking), consistent with the results for social disorder (Keizer et al., 2008). Therefore, in Experiment 2, participants were exposed to basic visual disorder or visual order pictures to explore whether basic visual disorder/order influenced the perceptual and conceptual processing style and to further support the results of Experiment 1. The hypothesis was the same as that in Experiment 1: Individuals’ perceptual and conceptual processing styles tend to be global or local after they experience orderliness and disorderliness primed by the basic visual order/disorder pictures used in Kotabe et al.’s study (see the illustration in Figure 1). In addition, we examined whether individuals’ needs for simple structure mediated the relationship between basic visual orderliness/disorderliness and types of cognitive processing.

### Methods

#### *Participants*

Fifty‐four healthy college students were paid to participate in Experiment 2. Data from two participants were discarded because the total target response time of the perceptual processing task was more than 2 *SD* from the mean. The data from the remaining 52 participants (34 females, age range: 18–22 years, *M*_age_ = 19.35 years, *SD* = 0.97 years) were used. The disorder priming condition included 25 participants’ data, and the order priming condition included 27 participants’ data.

#### *Apparatus and stimuli*

The apparatus was the same as that in Experiment 1. The visual order/disorder priming stimuli were the pictures used in the study by Kotabe et al. (2016b; Figure 1A).^2^ A separate group of 23 participants (11 females, age range: 17–19 years, *M*_age_ = 20.73 years, *SD* = 2.66) was first shown all 60 pictures (fixed size of 900 × 675 pixels) one by one and were made aware of the content and the order/disorder condition in the pictures. Then, they were asked to evaluate the order/disorder level of each picture on a 7‐point semantic differential scale anchored by the options *very disorderly* and *very orderly*. A paired *t* test revealed that the rating score for the disorderly pictures (*M* = 2.55, *SD* = 1.14) was significantly lower than that for the orderly pictures (*M* = 5.25, *SD* = 1.00), *t*(22) = −11.31, *p* < .001.

We also used the SAM assessment technique to measure the participants’ affective state after they viewed the basic visual disorder and order pictures. Paired *t* tests revealed that in the Pleasure dimension, the orderly pictures (*M* = 6.00, *SD* = 1.62) produced significantly higher pleasantness than the disorderly pictures (*M* = 4.52, *SD* = 1.56), *t*(22) = 3.82, *p* < .01. In the Arousal dimension, the arousal level of the orderly pictures (*M* = 5.74, *SD* = 1.98) was marginally significantly higher than that of the disorderly pictures (*M* = 5.04, *SD* = 1.94), *t*(22) = 1.86, *p* = .076. In the Dominance dimension, the dominance level of the orderly pictures (*M* = 5.69, *SD* = 1.63) was significantly higher than that of the disorderly pictures (*M* = 4.47, *SD* = 1.73), *t*(22) = 2.46, *p* < .05.

The stimuli of the Kimchi–Palmer figures task and the categorization task were the same as in Experiment 1.

### Design and procedure

Experiment 2 was also a between‐subjects design with two levels: a priming of the basic visual disorder picture condition and a priming of the basic visual order picture condition. The dependent variables and covariate variables were the same as in Experiment 1.

In Experiment 2, each participant sequentially completed the Kimchi–Palmer figures task and the categorization task using the E‐prime software.

The procedures of the Kimchi–Palmer figures task and the categorization task were similar to those in Experiment 1, except that the physical environment priming pictures in Experiment 1 were replaced with basic visual order/disorder pictures in Experiment 2.

### Results and Discussion

#### *Perceptual processing style*

An independent *t* test analysis between the disorder priming condition and the order priming condition was conducted separately for the response time and global target scores, and the Greenhouse–Geisser correction was applied to compensate for possible effects of nonsphericity in the measurements. The statistical analysis showed that the average response time in the disorder priming condition (*M* = 1758.11 ms, *SD* = 973.73) was significantly longer than that in the order priming condition (*M* = 1267.26 ms, *SD* = 572.48), *t*(50) = 2.24, *p <* .05, Cohen's *d* = 0.62. The statistical analysis of the global target scores revealed a significant difference between the two conditions *t*(50) = −2.85, *p <* .01, Cohen's *d* = 0.80, in which the average global target score in the disorder prime condition (*M* = 18.04, *SD* = 10.94) was significantly smaller than that in the order prime condition (*M* = 26.85, *SD* = 11.35). Figure 2 depicts the response time and global target scores.

The Pearson correlation analysis revealed no significant correlations between the global target scores and habits and the PNS score (*r*_habit_ = .10; *r*_PNS_ = −.18). The univariate covariance analysis of the global target scores revealed a significant difference between the two conditions, *F*(1, 48) = 6.61, *p <* .05, $\eta_{p}^{2}=.12$, in which the average global target score in the disorder prime condition (*M* = 18.39, *SD* = 10.94) was significantly smaller than that in the order prime condition (*M* = 26.52, *SD* = 22.61). This finding indicated that the participants’ habits and degrees of PNS did not influence their perceptual processing, similar to Experiment 1.

#### *Conceptual processing style*

A two‐way repeated‐measures ANOVA with prime type (disorder vs. order) as the between‐subjects factor and exemplar type (typical exemplar vs. moderate exemplar vs. atypical exemplar) as the within‐subject factor was conducted. The main effect of priming type and the interaction effect did not approach statistical significance. The main effect of exemplar type was significant, *F*(2, 100) = 90.65, *p <* .001,$\eta_{p}^{2}=.65$, with higher rating scores for the typical exemplar words (*M =* 8.59, *SD =* 2.43) than for the moderate exemplar words (*M* = 6.69, *SD* = 1.89) and the atypical exemplar words (*M* = 3.61, *SD* = 1.73), *p*s < .001. Figure 3 depicts the rating scores of the exemplar words.

The Pearson correlation analysis revealed no significant correlations between the exemplar word scores and habits and the PNS score. We used the scores for the PNS Scale and the habits questionnaire as covariate variables. The multivariate covariance analysis revealed no main effects of picture priming type and exemplar type or any interaction effects, which indicated that participants’ habits and degrees of PNS did not influence their conceptual processing, similar to Experiment 1.

At the perceptual processing level, the results support Hypothesis 1 and are consistent with the findings of Experiment 1: Basic visual order pictures lead to global perceptual processing (higher global target scores), whereas basic visual disorder pictures lead to local perceptual processing. It might also be that the higher positive pleasantness of the orderly pictures contributes to global perceptual processing. However, at the conceptual processing level, the participants’ types of processing were similar after they viewed the disorderly and orderly basic visual pictures; therefore, neither Hypothesis 2 nor Hypothesis 3 was supported. Similar to Experiment 1, the participants’ PNS and their individual orderly or disorderly living habits did not influence their global or local perceptual or conceptual processing.

## Experiment 3: Orderliness/disorderliness experiences primed by imagining an orderly/disorderly environment

In Experiment 3, we manipulated imagination of the physical environment to prime the participants’ experience of disorderliness/orderliness. We expected that imagining order/disorder would further support the hypothesis of Experiments 1 and 2 that individuals’ perceptual processing styles tend to be global or local after they experience orderliness and disorderliness and further extend to the conceptual processing level. Furthermore, we examined whether individuals’ need for simple structure mediates the relationship between basic visual orderliness/disorderliness and types of cognitive processing.

### Methods

#### *Participants*

Seventy college students were paid to participate in Experiment 3. Data from seven participants (three from the disorder priming condition and four from the order priming condition) were discarded because most of the response data were not recorded by the E‐prime software. The data from the remaining 63 participants (45 females, age range: 18–22 years, *M* = 19.63 years, *SD* = 0.83) were used. The disorder priming condition included 34 participants’ data and the order priming condition included 29 participants’ data.

#### *Apparatus and stimuli*

The apparatus was the same as in Experiment 1. We set up one disorderly room and one orderly room and then took photographs of these two rooms (see Figure 1). A separate group of 30 participants was first shown the two pictures (fixed size of 600 × 398 pixels) one by one and were made aware of the content and the order/disorder of the pictures. Then, they were asked to evaluate the level of order/disorder for each picture on a 7‐point Likert scale (1 = *totally disorderly*, 7 = *mostly orderly*) considering the entire range of the pictures. A paired *t* test revealed that the rating score of the degree of disorder for the disorderly pictures (*M* = 2.07, *SD* = 0.98) was significantly lower than that for the orderly pictures (*M* = 5.90, *SD* = 0.99), *t*(29) = −14.12, *p* < .001.

We also used the SAM assessment technique to measure the participants’ affective state after they viewed the basic visual disorder and order pictures. Paired *t* tests revealed that in the Pleasure dimension, the orderly pictures (*M* = 6.67, *SD* = 1.60) produced significantly higher pleasantness than the disorderly pictures (*M* = 3.60, *SD* = 1.61), *t*(29) = 7.11, *p* < .001. In the Arousal dimension, the arousal level of the orderly pictures (*M* = 6.27, *SD* = 2.05) was marginally significantly higher than that of the disorderly pictures (*M* = 4.50, *SD* = 1.96), *t*(29) = 3.04, *p* < .01. In the Dominance dimension, the dominance level of the orderly pictures (*M* = 6.20, *SD* = 2.05) was significantly higher than that of the disorderly pictures (*M* = 4.77, *SD* = 2.03), *t*(29) = 2.30, *p* < .05.

Each participant was asked to view the disorderly or orderly room picture and then imagine themselves in that environment.

The Kimchi–Palmer figures task and the categorization task were similar to those in Experiment 1, except that no disorder/order priming picture was presented.

#### *Design and procedure*

Experiment 3 was a between‐subjects design with two levels: imagining a disorderly environment condition and imagining an orderly environment condition.

For the Kimchi–Palmer figures task, there were eight small blocks. In each block, the participants first viewed a disorderly or orderly environment picture and were asked to imagine themselves in the environment within 30 s. Then, the participants were asked to perform six trials of Kimchi–Palmer figure judgment. Each trial began with a central fixation cross (angle 0.8°) for 2000 ms, which was followed by a Kimchi–Palmer figure. The participants were asked to choose which comparison figure was more similar to the target figure by pressing the “F” or the “J” button as quickly as possible. The next trial began after a blank screen was presented for 1000 ms. After six trials, the participants were asked to view the disorderly/orderly picture and imagine it again. In total, the participants completed 48 experimental trials in which 24 disorderly (or orderly) priming pictures were each presented twice and randomly.

The procedure of the categorization task was similar to that of the Kimchi–Palmer figures task, except that after viewing disorderly/orderly pictures and imagining that environment, the participants were asked to rate exemplar words in a category on a 10‐point scale. There were three small blocks. In total, the participants completed 24 experimental trials in four categories in which 12 exemplar words were presented twice.

### Results and discussion

#### *Perceptual processing style*

An independent *t* test analysis between the disorderly priming condition and the orderly priming condition was conducted separately for the response time and global target scores, and the Greenhouse–Geisser correction was applied to compensate for possible effects of nonsphericity in the measurements. The statistical analysis revealed no significant difference in the average response time between the disorderly priming condition (*M* = 1335.07 ms, *SD* = 768.80) and the orderly priming condition (*M* = 1303.48 ms, *SD* = 551.47), *t*(61) = 0.18, *p* > .05. The statistical analysis of the global target scores revealed a significant difference between the two conditions, *t*(61) = −2.75, *p <* .01, Cohen's *d* = 0.70, in which the average global target score in the disorderly prime condition (*M* = 19.88, *SD* = 12.26) was significantly smaller than that in the orderly prime condition (*M* = 28.93, *SD* = 13.66). Figure 2 depicts the response times and global target scores.

The Pearson correlation analysis revealed no significant correlations between the global target scores and habits and the PNS score (*r*_habit_ = −.051; *r*_PNS_ = −.058). The univariate covariance analysis of the global target scores revealed a significant difference between the two conditions, *F*(1, 59) = 7.29, *p <* .01, $\eta_{p}^{2}=.11$, in which the average global target score in the disorderly prime condition (*M* = 19.89, *SD* = 12.26) was significantly smaller than that in the orderly prime condition (*M* = 28.92, *SD* = 13.66). This finding indicates that the participants’ habits and degrees of PNS did not influence their perceptual processing, similar to Experiments 1 and 2.

#### *Conceptual processing style*

A two‐way repeated‐measures ANOVA was conducted with prime type (disorder vs. order) as the between‐subjects factor and exemplar type (typical exemplar vs. moderate exemplar vs. atypical exemplar) as the within‐subject factor. The main effect of priming type and the interaction effect did not approach statistical significance. The main effect of exemplar type was significant, *F*(2, 122) *=* 269.70, *p <* .001, $\eta_{p}^{2}=.82$, with higher rating scores for the typical exemplar words (*M = 9.20*, *SD = 1.86*) than for the moderate exemplar words (*M = 6.98*, *SD = 1.71*) and the atypical exemplar words (*M = 3.34*, *SD = 1.44*), *p*s < .001. Figure 3 depicts the rating scores of the exemplar words.

The Pearson correlation analysis revealed no significant correlations between the exemplar word scores and habits and the PNS score. We further used the scores of the PNS Scale and the habit questionnaire as covariate variables. The multivariate covariance analysis revealed no main effect of picture priming type (*p* > .05). A high main effect of exemplar type, *F*(2, 118) = 6.46, *p <* .05, $\eta_{p}^{2}=.10$, was observed, with higher rating scores for the typical exemplar words (*M = 9.02*, *SD = 2.68*) than for the moderate exemplar words (*M = 6.98*, *SD = 2.13*) and the atypical exemplar words (*M = 3.46*, *SD = 1.67*), *p*s < .001. These results revealed that participants’ habits and the degrees of their PNS did not influence their conceptual processing.

In Experiment 3, at the perceptual processing level, the results supported Hypothesis 1 and were consistent with the findings of Experiments 1 and 2: Individuals’ perceptual processing styles tend to be local after they experience disorderliness, whereas they tend to be global after they experience orderliness. It might also be that the higher positive pleasantness of the orderly pictures contributes to global perceptual processing. However, at the conceptual processing level, the processing is similar after viewing the disorderly and orderly basic visual pictures; therefore, neither Hypothesis 2 nor Hypothesis 3 was supported. Similar to Experiments 1 and 2, the participants’ PNS scores and their individual habits of orderly or disorderly living did not influence the types of global and local perceptual and conceptual processing.

### General discussion

In the current study, we used three behavioral experiments to examine whether people's perceptual and conceptual processing (global vs. local) was influenced by a disorderly or orderly situation. We asked the participants to perform a typical Kimchi–Palmer figures task (perceptual processing task) or a categorization task (conceptual processing task) with primed disorderly or orderly physical environmental pictures (Experiment 1), basic visual pictures (Experiment 2), and imagining a real environment (Experiment 3). The results revealed that in all of the above operations, orderly experiences led to global perceptual processing, whereas disorderly experiences led to local perceptual processing. This processing style was not influenced by the participants’ daily habits or their preference for the need for structure. However, this difference in the perceptual processing level was not found at the conceptual processing level.

The results of the three experiments supported Hypothesis 1: In contrast to an orderly situation, a disorderly situation generated local perceptual processing. This finding indirectly supports the previously proposed broadening function of positive affect and the narrowing function of negative affect. Positive mood is associated with greater global and holistic processing (i.e., seeing the forest before the trees) than local processing (i.e., seeing the trees before the forest; Basso et al., 1996; Gasper & Clore, 2002). In the current study, by measuring participants’ affective state with the SAM assessment, we found that participants experienced lower pleasantness in response to the disorderly pictures and higher pleasantness in response to the orderly pictures. Bradley and Lang (1994) proposed that the “rating of pleasure reflect[s] one's tendency to approach a stimulus, whereas displeasure reflects a tendency to withdraw, escape, or otherwise terminate the encounter” (p. 57). This is in accordance with the explanation of the cognitive tuning model (Clore et al., 2001; Gasper & Clore, 2000; Schwarz, 1990; Schwarz & Bless, 1991; Schwarz & Clore, 1996). A positive mood signals that the world is safe and that goals are not threatened; in safe situations, individuals become more agreeable to risk‐taking and adopt a more heuristic/global approach. In contrast, a negative mood may convince individuals that their world is threatening and that their goals are compromised. In addition, Kotabe (2014) noted that disorderliness is related to randomness, whereas orderliness is associated with regularity. Using a series of implicit‐association tests and variants of the affective priming paradigm, independent of whether the task requires participants to focus on symmetry, evidence has shown that visual symmetry is linked to positive affect, namely, the experience of pleasantness (Bertamini, Makin, & Rampone, 2013; Pecchinenda, Bertamini, Makin, & Ruta, 2014). Indeed, the order stimuli used in our experiments were approximately symmetrical, and the disorder stimuli were random. Therefore, it may be that the lower feeling of pleasantness in response to disorderly pictures leads to an avoidant tendency, which is reflected by local perceptual processing, whereas the higher feeling of pleasantness in response to the orderly pictures is reflected by global perceptual processing.

The above interpretation or speculation regarding the lower pleasantness of disorderliness is also in accordance with the “world‐is‐random” (WIR) theory to some extent. From a purely cognitive perspective, Kotabe (2014) proposed the WIR theory, which can also explain the local perceptual processing of disorderly situations (Kotabe, 2014). The WIR proposes that perceiving disorder primes randomness‐related concepts (e.g., chance, luck), which may lead people to (accurately) believe that they have less control or are losing personal control. The sense of losing control is threatening, and this threat depletes cognitive resources (Inzlicht & Kang, 2010), which has a variety of detrimental affective consequences. Therefore, the WIR may explain the above‐mentioned consequence of unpleasantness that is related to the disorderly environment. That is, disorderliness makes people feel that the world is random, and they face the threat of losing their sense of self‐control and gain unpleasant affect, ultimately narrowing the perceptual processing to the local type.

By measuring participants’ affective state with the SAM assessment, we also found that in the arousal dimension, the arousal level of the orderly pictures was significantly higher than that of the disorderly pictures in all three experiments. One study provided a complex picture showing that symmetrical/regular patterns are more closely associated with arousal than random patterns (Bertamini et al., 2013). However, earlier studies argued that arousal increases with complexity, and random patterns are more complex than symmetrical patterns (Berlyne, 1970; Tyler, 1995; Wagemans, 1995), so they should theoretically be more arousing. The results of the current study indirectly demonstrate that symmetrical/regular patterns induce higher arousal than random patterns. However, unlike valence, arousal is not positive or negative, and it can be linked with either negative or positive states and with either avoidance or approach behavior (Bertamini et al., 2013). Therefore, the underlying impact of arousal on order and disorder perception must be further explored.

Another interpretation of the results of perpetual processing styles involves processing fluency. Fluency can be defined as the experienced ease of processing a stimulus, and increased processing fluency is assumed to elicit mild levels of positive affect. Dijkstra et al. (2014) found that participants using a global processing style (measured by the Navon letter task) had more experiences of processing fluency than participants using local processing (Dijkstra et al., 2014). Kotabe (2014) and Kotabe et al. (2016b) proposed that perceived disorder might be cognitively processed more disfluently than perceived order since disordered stimuli were more redundant and conveyed more information than ordered stimuli. These aspects could render the viewing of visually disordered stimuli more cognitively demanding than the viewing of visually ordered stimuli at a high processing level. Bertamini et al. (2013) proved that randomness patterns have lower perceptual fluency than regular/symmetry patterns. Our recently published event‐related potential study examined the neural signals of the disorderly and orderly pictures used in the current Experiments 1 and 2 and supports this assertion that orderly perception is more fluent than disorderly perception (Li et al., 2019). Therefore, on the basis of the above findings, the increased processing fluency generated from the primed orderly pictures may lead to participants’ choice of global perceptual processing, whereas the disfluency experienced by the primed disorderly pictures supports participants’ choice of local perceptual processing.

According to prominent views in cognitive psychology, high‐level cognition derives from and is connected to perception (e.g., Barsalou, 2008; Friedman & Förster, 2008). However, beyond our three hypotheses, we did not find that the impacts of disorderliness and orderliness on perceptual processing level spilled over to the conceptual processing level; that is, the effect of disorderliness versus orderliness is limited to the perceptual processing level. One possible reason for these null results for conceptual processing might be that the disorderly and orderly pictures primed in the three experiments did not approach the higher semantic processing level, or the degree of semantic processing level was not apparent. Another possibility involves the sensitivity of the categorization task, which was an inclusive/exclusive categorization task. The four categories of words and the 12 exemplar words were familiar in daily life and might not be affected by the situation. In future studies, disorderly and orderly manipulations should be established to approach the semantic level to induce disorderliness and orderliness. For example, Heintzelman et al. (2013) manipulated perceived disorder by: (a) presenting people with pictures of seasons in temporal sequence (e.g., autumn, winter, spring, summer; order manipulation) or presenting them in random sequence (e.g., winter, autumn, summer, spring; experiments 1 and 2; disorder manipulation); or (b) presenting people with semantic triads (i.e., remote association test items) that were either coherent (e.g., “falling, actor, dust”; common association: star) or incoherent (e.g., “belt, deal, nose”; experiments 3 and 4; Heintzelman et al., 2013). These operations considered semantic/conceptual processing. On the other hand, other conceptual processing tasks, such as creative or analytic tasks, could be used. For example, participants were asked to generate the most unusual exemplar they could think of for a number of categories (e.g., birds, colors, fruits; Friedman et al., 2003) or had to find a creative title for a cartoon and generate unusual uses for a brick (Friedman & Förster, 2001).

Two limitations should be noted. One involves the affective and processing fluency explanations for the impact of disorderly versus orderly cues on global/local perceptual processing. In the current study, it is difficult to determine or disentangle whether affective valence, processing fluency, or both cause the effect of disorderliness versus orderliness on global versus local processing, especially at the perceptual level. Previous research has shown that fluency and affective reactions are not independent and that fluency reinforces affective reactions (Dijkstra et al., 2014). Hence, further research is required to disentangle the role of processing fluency and affective reaction in perceptual and conceptual processing. Second, before the normal modified perceptual or conceptual processing task, we controlled the disorderly and orderly priming pictures by measuring the participants’ affective reactions through the SAM technique, including the dimensions of pleasure, arousal, and dominance. However, we did not measure the participants’ affective reactions when the participants finished the modified perceptual or conceptual processing tasks due to the limited duration of each disorderly and orderly picture (e.g., 2000 ms in Experiments 1 and 2) and the limitation of the SAM technique. This might weaken the interpretation of Hypothesis 1 that disorderliness elicited negative affect, which led to perceptual processing, whereas orderliness triggered positive affect, which produced global perceptual processing from the positive or negative affect viewpoint, as we discussed in the introduction. Future investigations could verify participants’ emotional state when they perform perceptual or conceptual processing tasks in situations of disorderliness or orderliness. In addition, it is necessary to investigate the implicit associations between negative affect and disorderliness and between positive affect and orderliness.

To conclude, the findings of the current work imply that environmental orderliness and disorderliness not only functionally activate different psychological states and produce different kinds of behavioral outcomes but also change cognitive processing, or how we see and think about events and objects. Thus, these findings bridge several lines of research and shed light on a basic cognitive mechanism responsible for perceptions of order/disorder.

## Disclosure of conflict of interest

The author declares that the research was conducted in the absence of any commercial or financial relationships that could be construed as a potential conflict of interest.

## Notes

1 All images can be requested from Professor Hong Lu, Department of Psychology, Guangzhou University. Email: luhong@gzhu.edu.cn

2 All images can be downloaded in original resolution here: goo.gl/S8ShgT
